# Management of multiple drug hypersensitivity in a child with very severe aplastic anemia: a case report and therapeutic challenge

**DOI:** 10.3389/fped.2026.1758240

**Published:** 2026-02-17

**Authors:** Meihua Chen, Yi Deng, Pengyu Bin, Rong Gao, Nan Liao, Zhilin Zhou, Xin Huang, Jiaojiao Li, Jingsong Wang

**Affiliations:** 1 Department of Pharmacy, Chengdu Women’s and Children’s Central Hospital, School of Medicine, University of Electronic Science and Technology of China, Chengdu, Sichuan, China; 2 Department of Pharmacy, Guangyuan Central Hospital, Guangyuan, Sichuan, China

**Keywords:** aplastic anemia, carbapenems, drug hypersensitivity, levofloxacin, neutropenia

## Abstract

This work presents the case of a pediatric patient with very severe aplastic anemia (VSAA) who developed severe hypersensitivity reactions sequentially to a carbapenem antibiotic and subsequently to intravenous levofloxacin during an episode of severe neutropenia with fever. This case illustrates that serious drug hypersensitivity reactions can occur even in the context of profound immunosuppression. In managing this complex scenario, the Naranjo Algorithm was utilized to identify the culprit medications. When adjusting the anti-infective regimen, we carefully balanced the benefits and risks, ultimately selecting appropriate alternative antimicrobial agents. Furthermore, the patient tolerated a switch from intravenous to oral levofloxacin without further adverse reactions, leading to a successful discharge. This case highlights the need for heightened vigilance among clinicians and pharmacists regarding the potential for severe drug allergies in immunocompromised, critically ill patients. This case also aims to share practical experience, hoping to provide a reference for the challenging decision-making process of anti-infective therapy in this vulnerable population.

## 1 Introduction

Severe aplastic anemia (SAA) is a group of bone marrow failure (BMF) syndromes characterized by hypoplasia of bone marrow nucleated cells and a reduction in two or three peripheral blood cell lineages, and it is caused by immune-mediated destruction of hematopoietic cells (1). The annual incidence of SAA in China is 0.74 per 100,000 population, affecting all age groups with no significant difference in incidence between males and females. Its primary clinical manifestations, including anemia, bleeding, and infection, correspond to the consequences of cytopenia. Infection stands as the leading cause of death in SAA patients. Due to severely compromised bone marrow function, these patients often experience persistent neutropenia, which significantly increases their risk of invasive fungal infections and systemic bacterial sepsis (2).

In China, Gram-negative bacilli account for over 50% of pathogens identified in febrile neutropenia (FN) with bloodstream infections. Common Gram-negative isolates include *Escherichia coli*, *Klebsiella pneumoniae*, *Pseudomonas aeruginosa*, *Stenotrophomonas maltophilia*, and *Acinetobacter baumannii* (3). The rate of antimicrobial resistance among bloodstream isolates is higher than that from other sites. Compared to Western countries, China faces a relatively high and increasing incidence of carbapenem-resistant Enterobacteriaceae infections in the general population, representing a significant challenge in the management of FN. For high-risk patients with persistent neutropenia, such as those with SAA, appropriate and timely empirical therapy is critical. If risk factors for resistant bacterial infection are present, monotherapy with a carbapenem or combination therapy with an anti-pseudomonal β-lactam agent plus an aminoglycoside or fluoroquinolone is recommended (3).

With the extensive use of carbapenems in severe infections, reports of associated adverse reactions have increased accordingly. Beyond common gastrointestinal reactions, liver function abnormalities, and neurological symptoms, life-threatening events such as anaphylactic shock occasionally occur (4, 5). A drug-induced hypersensitivity reaction is a hyperacute, systemic response that progresses rapidly and poses an immediate threat to life (6, 7). Due to the rarity and unpredictability of anaphylaxis, which can potentially lead to fatal outcomes, it constitutes a medical emergency for healthcare professionals (6). Although epinephrine, corticosteroids, and antihistamines serve as standard treatments for severe hypersensitivity reactions, managing these events remains challenging. This is compounded by the rarity of severe hypersensitivity reactions to carbapenems, which occur in only 0.3% to 2.3% of cases (8), and the considerable individual variation in hypersensitivity manifestations.

In clinical practice, it is often assumed that children with severe immunodeficiency, such as profound neutropenia, possess a diminished immune response capacity and consequently face a lower risk of severe hypersensitivity reactions (9). Nonetheless, when an SAA patient developing a severe infection experiences a serious hypersensitivity reaction to carbapenem therapy, selecting an alternative among aminoglycosides or fluoroquinolones introduces significant risks, including nephrotoxicity or tendon rupture. Weighing the risks and benefits of therapeutic options, particularly in an immunocompromised host, presents a considerable clinical dilemma.

This work reports a case of a child with very severe aplastic anemia (VSAA) who developed severe hypersensitivity reactions during anti-infective therapy for FN and subsequent serious infection. A second hypersensitivity reaction occurred after adjusting the treatment regimen. This report discusses key considerations for identifying drug allergies and the rationale for adjusting anti-infective strategies, aiming to provide clinical pharmacists with valuable experience and to optimize the comprehensive management of such critically ill children.

## 2 Case presentation

A 13-year-and-5-month-old boy, 41.5 kg and 155 cm, presented with spontaneous skin ecchymosis and petechiae of unknown cause in December 2024. Initially distributed on both lower limbs, the lesions progressively spread over his entire body, accompanied by one episode of suspected melena. No fever or cough was reported, with occasional rhinorrhea. Ten days later, due to the progressive worsening of the ecchymosis and petechiae, he sought medical attention at a local hospital. Coagulation tests showed no significant abnormalities, but complete blood count revealed a white blood cell count (WBC) of 5.03 × 10⁹/L (reference range: 4.1–11 × 10⁹/L), a platelet count (PLT) of 3 × 10⁹/L (reference range: 150–407 × 10⁹/L), and a hemoglobin (Hb) level of 109 g/L (reference range: 129–172 g/L). Consequently, he was admitted to our hospital on January 6, 2025, with an initial diagnosis of “Thrombocytopenic Purpura.”

**Figure 1:**
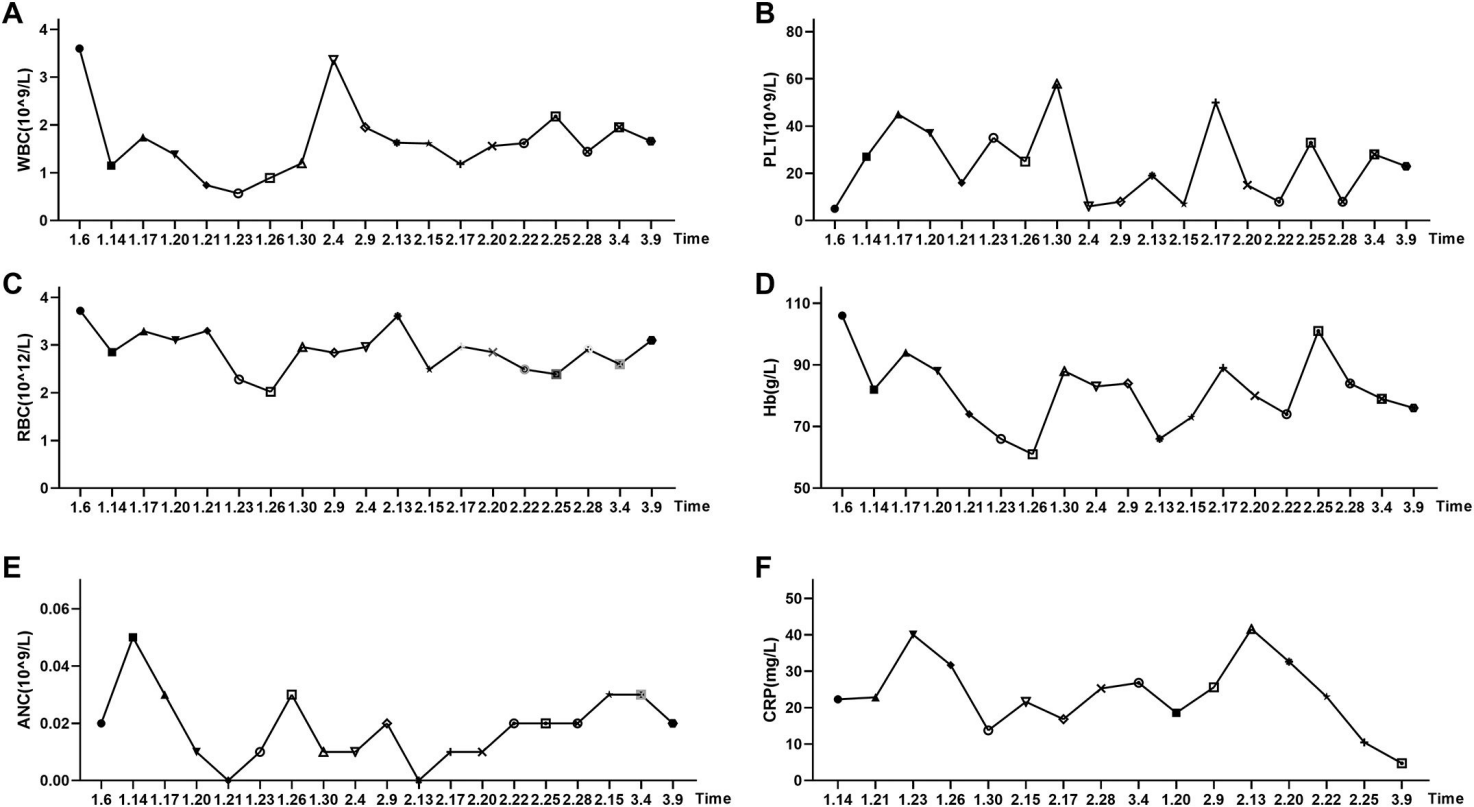
Changes in complete blood count parameters and C-reactive protein levels during the patient's hospitalization. **(A)** White blood cell count (WBC), reference range: 4.1–11.0 × 10⁹/L; **(B)** Platelet count (PLT), reference range: 150–407 × 10⁹/L; **(C)** Red blood cell count (RBC), reference range: 4.5–5.9 × 10^12^/L; **(D)** Hemoglobin (Hb), reference range: 129–172 g/L; **(E)** Absolute neutrophil count (ANC), reference range: 1.9–7.9 × 10⁹/L; **(F)** C-reactive protein (CRP), reference range: 0-10 mg/L.

Following admission, a bone marrow aspiration was performed. The bone marrow cytology results, available on January 8, 2025, indicated aplastic anemia. Bone marrow biopsy confirmed severely hypocellular marrow (<5% cellularity) with rare granulocytic and erythroid precursors and absent megakaryocytes, consistent with aplastic anemia. In conjunction with a PLT of 5 × 10⁹/L, an absolute neutrophil count (ANC) of 0.26 × 10⁹/L (reference range: 1.9–7.9 × 10⁹/L), and a red blood cell count (RBC) of 3.15 × 10^12^/L (reference range: 4.5–5.9 × 10^12^/L), the diagnosis was revised to SAA. Immunosuppressive therapy was initiated three days later, consisting of cyclosporine (100 mg q12 h po). However, three days after starting cyclosporine (on January 14), the patient developed a fever (peak 39 ℃) accompanied by dizziness and headache. Follow-up complete blood count and C-reactive protein (CRP) testing revealed a WBC of 1.15 × 10⁹/L and a CRP level of 22.3 mg/L (reference range: 0–10 mg/L) (Figure 1). The diagnosis was subsequently upgraded to VSAA. Cyclosporine was discontinued, and anti-infective therapy with imipenem-cilastatin sodium (500 mg q6 h) was started, alongside supportive care with intravenous immunoglobulin (10 g) and platelet transfusion.

By January 17, 2025, the patient continued to experience intermittent fever, dizziness, occasional headache, cough, and sputum production with slight blood streaking. Following a platelet transfusion, he developed urticarial erythema on his face and neck, which was considered a transfusion reaction. A head CT scan suggested a possible small subtentorial subarachnoid hemorrhage. Blood culture results returned negative. The patient received loratadine (10 mg qd po) and topical calamine lotion, leading to the resolution of the rash. After four days of imipenem-cilastatin therapy, the patient remained afebrile until January 21, when fever recurred (peak 38.5  ℃). Although his headache had resolved, he still had productive cough and developed new, densely distributed, pruritic, red rashes primarily on his face and trunk. Symptomatic treatment with intravenous compound glycyrrhizin and vitamin C was administered. Imipenem-cilastatin was continued, supplemented with oral oseltamivir for antiviral coverage and intravenous voriconazole for antifungal prophylaxis.

On the night of January 23, the patient's fever persisted (peak 39.1 °C) without chills or rigors. The generalized rash showed no significant improvement or worsening but remained pruritic. He subsequently developed facial and bilateral upper limb edema. During the febrile episode, his blood pressure dropped to a minimum of 88/48 mmHg and failed to stabilize adequately after fluid resuscitation. He required norepinephrine for hemodynamic support, alongside aggressive shock management including volume expansion, fluid replacement, and transfusions of packed red blood cells, platelets, and albumin. Consequently, he was transferred to the intensive care unit (ICU) for further monitoring and management.

In the ICU, anti-allergy treatment was continued. The antimicrobial regimen was adjusted, imipenem-cilastatin was switched to amikacin, and both voriconazole and oseltamivir were discontinued. The patient's rash gradually improved, fading to a dark red color without new lesions, and the peak body temperature decreased. After two days (on January 26), the rash had largely resolved, the edema had subsided, and his body temperature had normalized.

After remaining afebrile for three days (on January 29), fever recurred (peak 38 ℃). At this point, two sets of blood cultures flagged positive within 24 h, suggesting the presence of Gram-positive cocci. Empirical anti-infective therapy with linezolid (600 mg every 12 h) was subsequently initiated. On February 1, the blood culture identification returned as *Streptococcus mitis*, and drug susceptibility testing indicated sensitivity to linezolid. Given that both bottles of the blood cultures were positive and flagged positive rapidly, contamination was considered unlikely. Furthermore, after the addition of linezolid, the child's body temperature returned to normal within three days, and systemic symptoms of infection improved. Based on this comprehensive assessment, *Streptococcus mitis* was considered highly likely to be the causative pathogen for this febrile episode. The subsequent anti-infective regimen was maintained as linezolid in combination with amikacin for continued treatment. Following seven days of stable temperature (on February 8), low-grade fever recurred (peak 38.1 ℃), which slowly resolved with oral antipyretics. He had no cough, abdominal pain, new skin rashes, epistaxis, or gingival bleeding. Empirical antifungal coverage was enhanced with the addition of caspofungin, while linezolid and amikacin were continued. On February 14, the serum galactomannan (GM) test result was 1.74 (normal reference value <0.5), indicating a positive result.

**Figure 2:**
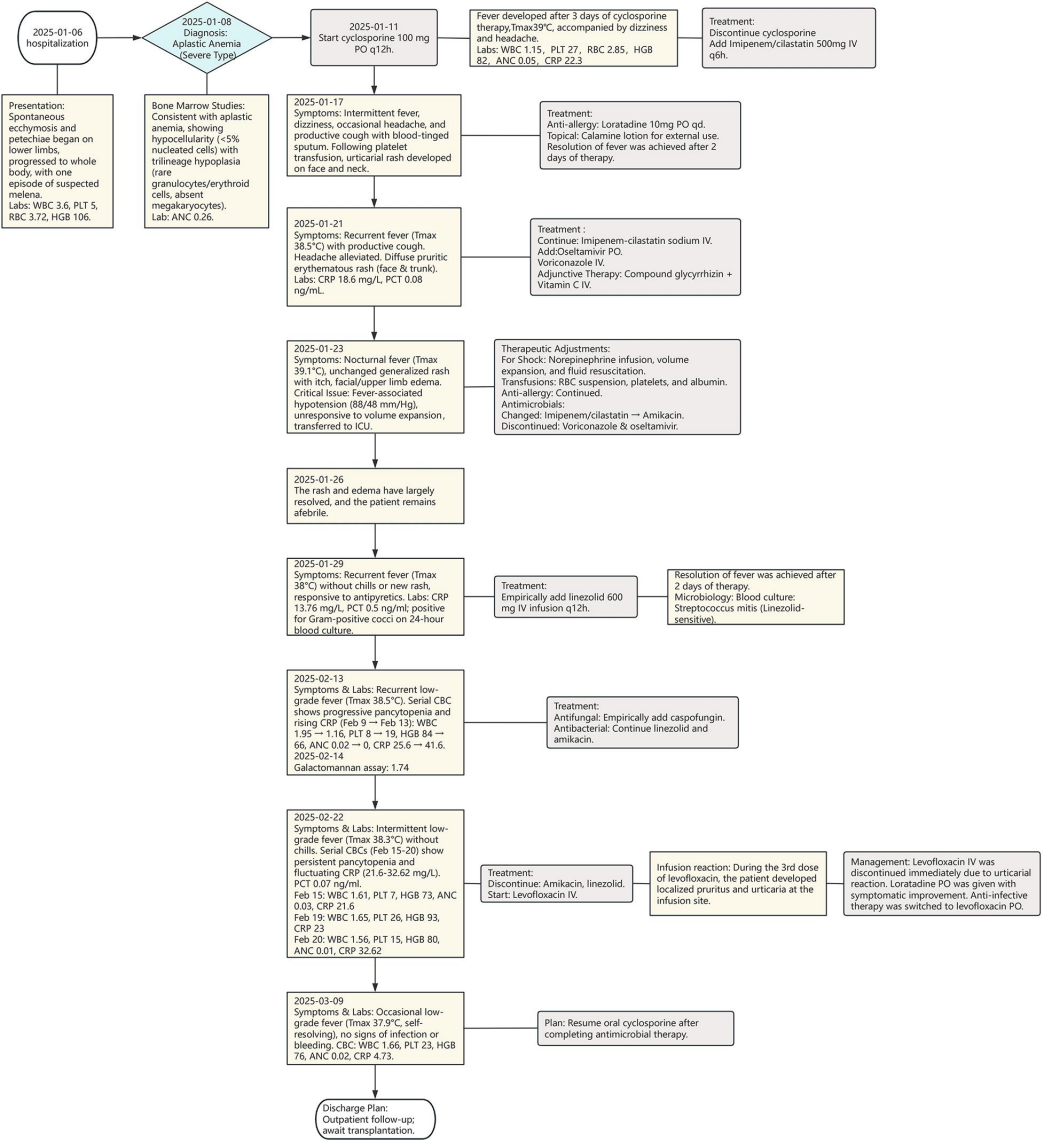
Detailed treatment timeline during the patient's hospitalization.

Intermittent low-grade fever (peak 38.3  ℃) persisted until February 22, without associated chills or rigors. At this point, amikacin and linezolid were discontinued, and intravenous levofloxacin was initiated for anti-infective therapy. On February 23, during the third infusion of intravenous levofloxacin, the patient developed pruritus at the infusion site, followed by the appearance of urticarial rash over his body within three minutes. Intravenous levofloxacin was immediately stopped, and oral loratadine was administered, leading to gradual rash resolution. As ongoing anti-infective therapy was still required, the regimen was switched to oral levofloxacin tablets, which were tolerated without any allergic manifestations. A repeat CBC on February 25 showed improvement. The patient experienced only occasional, self-resolving low-grade fever (max 37.9 ℃) with no apparent signs of active infection or bleeding tendency. Following the completion of antibacterial and antifungal therapy, cyclosporine oral solution was re-initiated. The patient was discharged for outpatient follow-up to await transplantation. A detailed timeline of the patient's clinical course and treatment is depicted in Figure 2.

## 3 Discussion

Drug-induced hypersensitivity reactions are triggered by the rapid release of a multitude of functionally diverse mediators, with histamine and platelet-activating factor identified as key drivers. Experimental models of anaphylaxis have established that the mechanism involves drug-specific IgE and IgG antibodies. These antibodies bridge the drug to Fc receptors on various cell types, including mast cells, neutrophils, platelets, basophils, macrophages, and monocytes, leading to cellular activation and mediator release (6). IgG antibodies can activate neutrophils and other cells expressing activating Fc-gamma receptors (Fc*γ*Rs), either directly inducing anaphylaxis or synergizing with IgE of the same specificity. Among circulating cells, platelets and neutrophils, given their high abundance, may play a dominant role in IgG-mediated pathways. In contrast, mast cells and basophils are more readily activated via IgE-dependent and MRGPRX2-dependent pathways, where even minimal IgE binding to Fc*ε*RI can trigger activation (6). Furthermore, studies suggest that allergen-specific IgE-producing B cells originate from mature B cells that initially produced allergen-specific IgG (10).

It is generally accepted that children with VSAA, characterized by pancytopenia, including deficiencies in platelets, neutrophils, and B lymphocytes, and an immunocompromised state, should theoretically be less susceptible to hypersensitivity reactions induced via classic IgE and IgG pathways. However, this patient experienced a severe drug hypersensitivity reaction. On the seventh day of imipenem-cilastatin sodium therapy, the child developed a delayed hypersensitivity reaction, primarily manifesting as a generalized rash accompanied by pruritus, fever, edema, and a drop in blood pressure. Norepinephrine was administered to elevate blood pressure, along with anti-shock measures including volume expansion and fluid resuscitation. However, blood pressure remained unstable, raising the possibility of anaphylactic shock. The clinical pharmacy team considered both infectious and drug-related causes for the rash and fever, raising vigilance for drug hypersensitivity syndrome. A thorough review of the patient's medication history assessed using the Naranjo Adverse Drug Reaction Probability Scale and cross-referenced with drug package inserts (Table 1). In summary, medications with Naranjo scores ≥5, which corresponds to a “probable” adverse drug reaction rating, included imipenem-cilastatin sodium, carbazochrome sodium sulfonate, and cyclosporine (in both capsule and oral solution formulations).

**Table 1:** Naranjo adverse drug reaction probability scale.

Question	Carbazochrome sodium sulfonate for injection	Methylprednisolone sodium succinate for injection	Brown mixture syrup	Compound pholcodine oral solution	Cyclosporine capsules	Cyclosporine oral solution	Imipenem and cilastatin sodium for injection
Are there conclusive research reports on this adverse reaction?	+1	+1	0	0	+1	+1	+1
Did the adverse reaction appear after the drug was administered?	+2	+2	+2	+2	+2	+2	+2
Did the adverse reaction improve when the drug was discontinued or a specific antagonist was administered?	+1	0	0	0	0	0	+1
Did the adverse reaction reappear upon rechallenge?	0	−1	0	0	0	0	0
Are there alternative causes that could have produced the reaction?	+2	+2	+2	+2	+2	+2	+2
Did the reaction reappear upon placebo readministration?	0	0	0	0	0	0	0
Was the drug detected in blood/body fluids at toxic concentrations?	0	0	0	0	0	0	0
For this particular patient, was there a positive correlation between the drug dosage and the severity of the adverse reaction?	0	0	0	0	0	0	0
Did the patient have a previous history of similar reactions to the same or similar drugs?	0	0	0	0	0	0	0
Was the adverse reaction confirmed by objective evidence?	0	0	0	0	0	0	0
Total Score	6	4	4	4	5	5	6

The Naranjo scale assessment results are categorized into four levels: ≤0 points indicates “doubtful”; 1–4 points indicates “possible”; 5–8 points indicates “probable”; and ≥9 points indicates “definite” causality.

Following the discontinuation of all suspected drugs and two days of symptomatic management, the rash began to fade and partially resolve, the edema subsided, and body temperature normalized. At this juncture, the patient remained severely neutropenic. According to risk stratification for FN and considering risk factors for resistant bacterial infection (3), he was classified as high-risk for resistant pathogens, given an anticipated duration of severe neutropenia (ANC <0.1 × 10⁹/L) exceeding 7 days and prior exposure to broad-spectrum antibiotics. With the infection site and causative organism still unidentified, intravenous empiric antibiotic therapy needed to provide coverage against *Pseudomonas aeruginosa* and other serious Gram-negative bacilli.

The emergence of a severe hypersensitivity reaction to first-line carbapenem therapy posed a significant challenge for subsequent anti-infective strategies. Due to the high cross-reactivity between imipenem and penicillin based on their shared bicyclic β-lactam ring structure, the use of imipenem should be avoided in patients with penicillin allergy. In contrast, aztreonam is a monobactam antibiotic, and its core structure is distinct from that of penicillins. Current evidence indicates that, aside from limited cross-reactivity with ceftazidime due to similar side chains, aztreonam generally exhibits no clinically significant cross-reactivity in patients allergic to penicillin (11, 12). Therefore, aztreonam serves as an important alternative therapeutic option for such patients (13, 14). Furthermore, the detection rate of carbapenem-resistant Gram-negative bacteria has been increasing annually. These bacteria produce diverse carbapenemases, with KPC (a serine β-lactamase) and NDM (a metallo-β-lactamase) being the most predominant types (15). Notably, aztreonam is stable against metallo-β-lactamases (such as NDM, IMP, and VIM), making it a crucial treatment option for infections caused by CRGNB producing these enzymes. However, it is ineffective against strains producing serine β-lactamases like KPC. As alternative agents, combinations of β-lactam antibiotics with fluoroquinolones or aminoglycosides may be considered. For pediatric patients, however, a careful assessment of potential benefits vs. risks is required. Amikacin, active against most Enterobacteriaceae (*E. coli, Klebsiella and Enterobacter*), *P. aeruginosa*, and other non-fermenters like *Acinetobacter* and *Alcaligenes species*, provides coverage against common pathogens in FN. Considering that the patient had experienced a severe allergic reaction and had a critical underlying condition requiring rapid initiation of effective therapy, the treatment team cautiously opted to continue anti-infective therapy with amikacin, an aminoglycoside antibiotic that lacks a β-lactam ring structure and carries a lower risk of drug eruption. During treatment, serum creatinine levels were monitored regularly to guard against its potential ototoxicity and nephrotoxicity.

Despite ongoing coverage for Gram-positive, Gram-negative, and fungal pathogens, the patient continued to experience intermittent fever, raising concerns about a new infection or a breakthrough infection with a resistant organism (resistant *E. coli, Klebsiella* and *Enterobacter*). Given the perceived risk of cross-reactivity among β-lactams and the assumed absence thereof with fluoroquinolones, the regimen was switched to intravenous levofloxacin, which offers broad coverage against most Gram-negative and Gram-positive bacteria, including aerobes and anaerobes.

During the third infusion of intravenous levofloxacin (approximately 3 min after initiation), the patient developed pruritus at the infusion site, followed by progressively worsening urticarial rash. Levofloxacin was immediately discontinued, and symptomatic treatment led to gradual rash resolution without recurrence. Based on a Naranjo Adverse Drug Reaction Probability Scale score of 5, the rash was considered a probable allergic drug reaction induced by the intravenous formulation of levofloxacin. Given the limited subsequent therapeutic options following this allergic reaction and the observed improvement in symptoms after discontinuing the intravenous formulation, the treatment team, after comprehensive assessment, decided to switch to oral levofloxacin tablets for continued treatment, provided the child's vital signs remained stable. This decision was primarily based on the following considerations: the limited availability of suitable alternative drugs; intravenous administration is more likely to trigger rapid and severe systemic reactions compared to oral administration (16), whereas in this instance, the rash was localized to the infusion site and relatively mild, and oral administration allows for slower absorption enabling immediate discontinuation should a reaction occur. Concurrently, the team implemented a rigorous monitoring plan, which included having resuscitation equipment readily available at the bedside and increased rounds by clinical pharmacists. Furthermore, prior to administering the medication, they fully communicated the therapeutic challenges and potential risks to the child's guardians and obtained informed consent to minimize risks to the greatest extent possible. Fortunately, no new rash appeared after switching to the oral formulation.

Immediate hypersensitivity reactions to levofloxacin are associated with both IgE-mediated mechanisms and direct activation of the mast cell-specific receptor MRGPRX2 (17–19). MRGPRX2 activation can induce rapid mast cell degranulation and release of histamine and other inflammatory mediators, precipitating a faster, more localized response (20, 21). Delayed hypersensitivity reactions, in contrast, are primarily T-cell mediated (22). Currently, diagnostic tools for confirming delayed-onset fluoroquinolone hypersensitivity are limited. Skin testing has poor sensitivity and specificity for diagnosing quinolone allergy (23). While the basophil activation test is considered a necessary adjunct and is more sensitive than skin testing (24), sits predictive value is suboptimal, and standardized commercial assays are lacking (24–26). The drug provocation test remains the only reliable tool to definitively confirm or exclude fluoroquinolone allergy (26). Drug provocation test may be considered after mild cutaneous reactions (26, 27), but carries inherent risks and is generally contraindicated following severe initial reactions, such as severe cutaneous adverse reactions.

For critically ill, immunocompromised patients like those with VSAA, managing secondary infections is inherently complex. This case underscores the necessity of maintaining a high index of suspicion for severe drug hypersensitivity reactions. In cases of anaphylactic shock, immediate administration of epinephrine is critical, followed by prompt adjustment of the medication regimen, diligently avoiding agents with potential for cross-reactivity.

## 4 Conclusions

This work reports the case of a pediatric patient with VSAA who developed hypersensitivity reactions to both a carbapenem antibiotic and intravenous levofloxacin during a period of severe neutropenia. This case demonstrates that severe drug hypersensitivity reactions can still occur even in profoundly immunocompromised children, and their underlying mechanisms may not solely depend on classical IgE/IgG-mediated pathways, thereby presenting significant challenges for clinical identification and management.

When first-line antimicrobial agents become unavailable due to hypersensitivity, selecting subsequent anti-infective regimens becomes exceptionally complex. This requires careful balancing of efficacy, potential adverse effects, and the risk of recurrent hypersensitivity reactions. The fact that this patient reacted to intravenous levofloxacin but tolerated its oral formulation suggests that the route of administration could be a critical factor influencing the clinical response. This observation provides valuable insights for medication management in children with a history of drug allergies.

In conclusion, for critically ill children, such as those with VSAA, vigilant monitoring for severe drug hypersensitivity is essential during aggressive anti-infective therapy. Should a reaction occur, immediate resuscitation and rapid assessment must be followed by the prompt identification of alternative regimens with no risk of cross-reactivity. This case offers important practical experience in clinical pharmacy regarding the recognition of drug hypersensitivity and the adjustment of anti-infective strategies for managing critically ill, immunocompromised patients with severe concurrent infections.

## Data Availability

The original contributions presented in the study are included in the article/Supplementary Material, further inquiries can be directed to the corresponding authors.
